# Gradient Multilayer Design of Ti_3_C_2_T_*x*_ MXene Nanocomposite for Strong and Broadband Microwave Absorption

**DOI:** 10.1002/smsc.202200018

**Published:** 2022-05-21

**Authors:** Yajun Zhang, Long Pan, Peigen Zhang, ZhengMing Sun

**Affiliations:** ^1^ Key Laboratory of Advanced Metallic Materials of Jiangsu Province School of Materials Science and Engineering Southeast University Nanjing 211189 P. R. China

**Keywords:** broadband microwave absorption, gradient structures, impedance matching, microwave absorption

## Abstract

High‐performance electromagnetic (EM) absorbing materials are in urgent need due to severe EM pollution caused by fast development of communication technology and electronic devices. Gradient structure benefits EM absorbing performance. However, the relevant study is very limited, and the relationship between component layers and overall performance is unclear. Herein, a gradient structure containing impedance matching layer, lossy layer, and reflective layer is built via repeated casting‐drying method using Ti_3_C_2_T_*x*_ MXene and polyvinyl alcohol. With optimized gradient structure, the minimum reflection loss (RL) reaches −74.8 dB with effective absorbing bandwidth (EAB) covering the whole X‐band. The good absorbing performance is ascribed to the good impedance matching, which is derived from the stepwisely increased permittivity of each layer. Moreover, it is found that RL reaches the lowest value when lossy layer permittivity is close to the average permittivity of impedance matching layer and reflective layer. In addition, the decrease of lossy layer permittivity causes the decrease of total thickness of the gradient structure. This work demonstrates the effectiveness of gradient structure toward strong and broadband microwave absorption and provides rules for designing proper gradient structures to satisfy different requirements.

## Graphical abstract

A three‐layer gradient structure is constructed using Ti_3_C_2_T_*x*_ MXene and polyvinyl alcohol for high‐performance microwave absorption. With optimized structure, the minimum reflection loss reaches −74.8 dB with effective absorbing bandwidth covering the whole X‐band. The outstanding performance is ascribed to the best overall impedance matching. General design guideline for gradient structure is also proposed in this research.

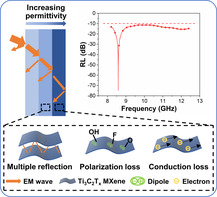

## 1 Introduction

With the fast development of communication technology and electronic devices, electromagnetic (EM) pollution has been more severe than ever, and rises to a primary concern since EM radiation is harmful to human health and a potential risk to cause malfunction of electronics, especially high‐sensitivity electronics.^[^ ^1^, ^2^, ^3^, ^4^ ^]^ To address these problems, large efforts have been devoted to develop EM absorbing materials with light weight, thin thickness, strong absorbing ability, and broad absorbing bandwidth.^[^ ^5^, ^6^, ^7^, ^8^, ^9^ ^]^

EM absorbing materials require good impedance matching to allow EM wave going inside of material and strong lossy ability to dissipate it. Unfortunately, good impedance matching and strong lossy ability are a contradictory pair. Good impedance matching usually means weak lossy ability, and strong lossy ability often represents poor impedance matching. Therefore, it is crucial to find the balance. Gradient structure design is a good idea to balance these two factors. In a gradient structure, the outer layer, which reacts with EM wave in the first place, is designed as low permittivity to offer good impedance matching with air, while the inner layer is designed as medium permittivity or high permittivity to consume or reflect penetrated EM wave. Consequently, gradient structure improves the EM absorbing ability of materials.^[^ ^10^, ^11^, ^12^, ^13^, ^14^, ^15^ ^]^

However, the study on gradient structure is very limited. Current researches mostly focus on the microstructure design of EM absorbing agent, which is homogeneously mixed with paraffin or resin to form a uniform EM absorber.^[^ ^1^, ^6^, ^7^, ^16^, ^17^, ^18^, ^19^, ^20^, ^21^ ^]^ For example, poly‐pyrrole microspheres were wrapped by polyaniline to form the core‐shell microstructure, but simply mixed with paraffin to build a uniform absorber.^[^ ^20^ ^]^ A novel seed‐germination‐like microstructure was designed to enhance interface polarization and magnetic loss, which was also blended with paraffin to form an even EM absorber.^[^ ^16^ ^]^ Even though these ingenious microstructure designs are indeed effective, it is supposed that the microwave absorbing performance might be further improved with additional gradient structure design.

Additionally, in the limited literature on gradient structure, the number of component layers is usually large. For example, a seven‐layer gradient structure was constructed by overlaying MXene/polymer composite films with increasing MXene content.^[^ ^10^ ^]^ This would cause huge amount of calculation to optimize the thickness of each layer, which is not friendly for designing a new gradient structure. Additionally, large number of layers can lead to very complex fabrication process since they are built layer by layer. Moreover, the relationship between permittivity of component layers and the performance of the gradient structure has never been reported as far as we know since the permittivity of each layer is fixed.

MXene, a new family of 2D material, has shown great potential in microwave absorbing field due to the large surface area and metal‐like conductivity.^[^ ^22^, ^23^, ^24^ ^]^ The outstanding conductivity endows MXene with strong lossy ability, but at the same time, very poor impedance matching. Therefore, it is meaningful to improve impedance matching of MXene via gradient structure design.

Herein, we report a gradient structure with only three layers for strong and wideband EM absorption. The three layers are named as impedance matching layer, lossy layer, and reflective layer, respectively, with elevated permittivity. Each layer is composed of Ti_3_C_2_T_*x*_ MXene and polyvinyl alcohol (PVA). With optimized gradient structure, the minimum reflection loss (RL) reaches −74.8 dB and at the same time effective absorbing bandwidth (EAB) covers the whole X‐band (8.2–12.4 GHz). By changing MXene concentration of the lossy layer, it is found that the RL of the gradient structure could achieve the lowest value when the permittivity of lossy layer is close to the average permittivity of impedance matching layer and reflective layer. In addition, the smaller the permittivity of lossy layer, the thinner the total thickness of the gradient structure. This work provides a template for realizing strong and wideband EM absorbing material through a simple three‐layer gradient structure and provides some general rules to design suitable gradient structures to satisfy different applications.

## 2 Results and Discussion

### 2.1 Microstructure and Morphology of Ti_3_C_2_T_x_/PVA Composite Films

Ti_3_C_2_T_*x*_ MXene nanosheets are derived from etching of Ti_3_AlC_2_ MAX phase. Phase and morphology evolution from MAX to MXene are shown in Figure S1, Supporting Information. The disappearance of the main peak of Ti_3_AlC_2_ MAX at 2*θ* = 39° and the emergence of the main peak of Ti_3_C_2_T_*x*_ MXene at 2*θ* = 6.2° demonstrate the successful etching of MAX phase. Figure S1b, Supporting Information, shows the compactly stacked layer structure of MAX phase. Figure S1c,d, Supporting Information, exhibits the scanning electron microscope (SEM) and transmission electron microscope (TEM) images of Ti_3_C_2_T_*x*_ MXene, indicating the thin and flexible nanosheet morphology. Figure S1e, Supporting Information, is the corresponding selected area electron diffraction (SAED), which demonstrates that the nanosheet is single crystal with a hexagonal atomic arrangement. In addition, a lattice space of 0.26 nm is measured in the high‐resolution TEM (HRTEM) image (Figure S1f, Supporting Information), which corresponds to the (0$\bar{1}$10) lattice plane of Ti_3_C_2_T_*x*_ MXene.^[^ ^25^ ^]^ In general, Ti_3_C_2_T_*x*_ MXene is successfully prepared.

PVA is selected as the matrix among various polymers mainly because a) PVA is a common water‐soluble polymer. Since Ti_3_C_2_T_*x*_ MXene has great hydrophilicity, it can be homogeneously dispersed into water and form colloid. Therefore, it is easy to uniformly mix MXene with polymer if they are both dissolved in water. b) The hydroxyl of MXene and PVA could form hydrogen bond, further promoting the homogeneously blending of MXene and PVA in microscope. c) Mechanical strength of PVA film is good, which makes it possible to fabricate strong PVA matrix nanocomposite.

**Figure 1:**
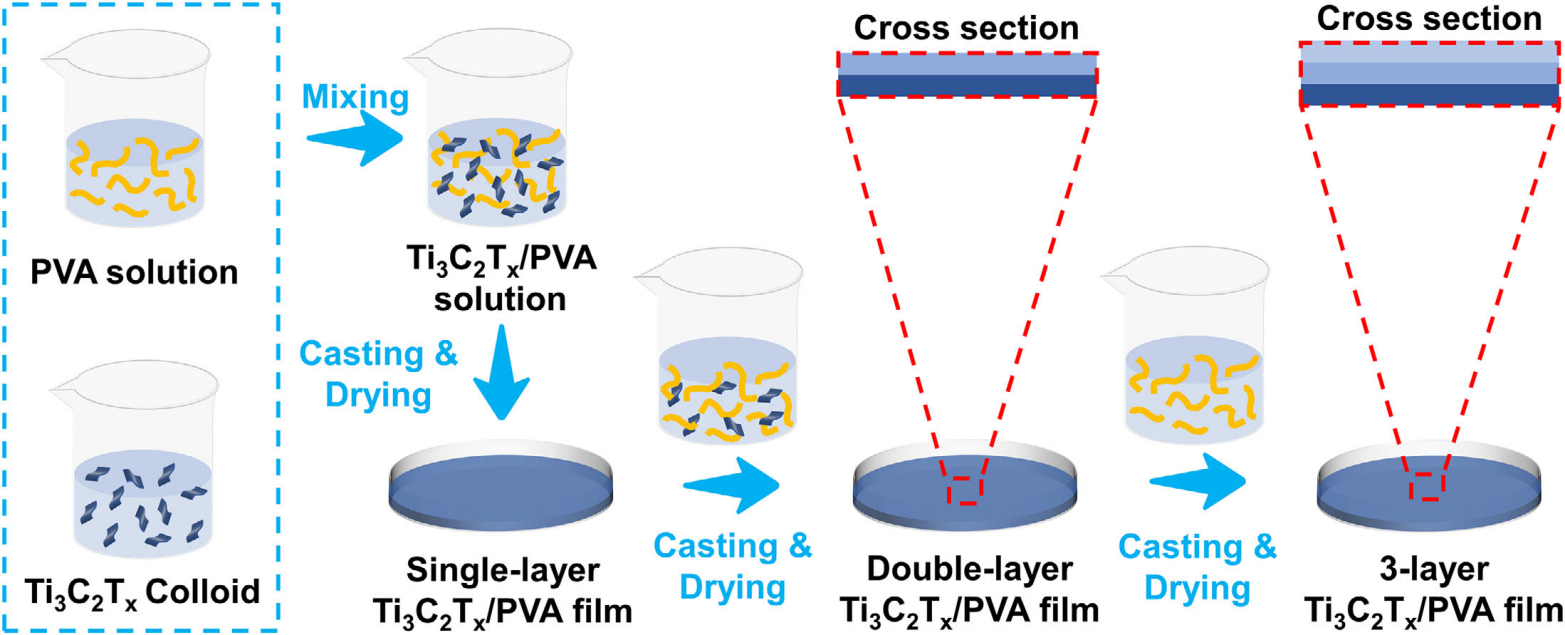
Illustration of fabrication process of single and multilayer Ti_3_C_2_T_*x*_/polyvinyl alcohol (PVA) composite films.

Ti_3_C_2_T_x_/PVA composite films are fabricated via a simple solution blending method as illustrated in **Figure** **1**. Different amount of Ti_3_C_2_T_*x*_ MXene colloid and PVA solution are mixed and stirred. Then, the mixtures are cast into plastic Petri dishes and dried. The single‐layer films are obtained from peeling off the films from the dishes. For convenience, the single‐layer films are labeled as 1% MXene, 3% MXene, 5% MXene, 7% MXene, and 10% MXene corresponding to Ti_3_C_2_T_*x*_ to PVA mass ratios of 1:100, 3:100, 5:100, 7:100, and 10:100, respectively. Regarding to the synthesis of a three‐layer gradient film, Ti_3_C_2_T_*x*_ and PVA mixture with a mass ratio of 10:100 is first cast into a Petri dish and dried. Then, Ti_3_C_2_T_*x*_ and PVA mixture with a mass ratio of 5:100 is cast into the same dish and dried. Finally, pure PVA solution is cast into that dish and dried. For short, this three‐layer gradient film is labeled as M10‐5‐0. Similarly, M10‐3‐0 means a three‐layer gradient structure with Ti_3_C_2_T_*x*_ to PVA mass ratio of 10:100 as the bottom layer and 3:100 as the middle layer and pure PVA as the top layer. Figure S2, Supporting Information, shows the photographs of 10% MXene as an example. The obtained films look smooth and exhibit good flexibility and toughness, which is benefit from the good mechanical property of PVA matrix.

**Figure 2:**
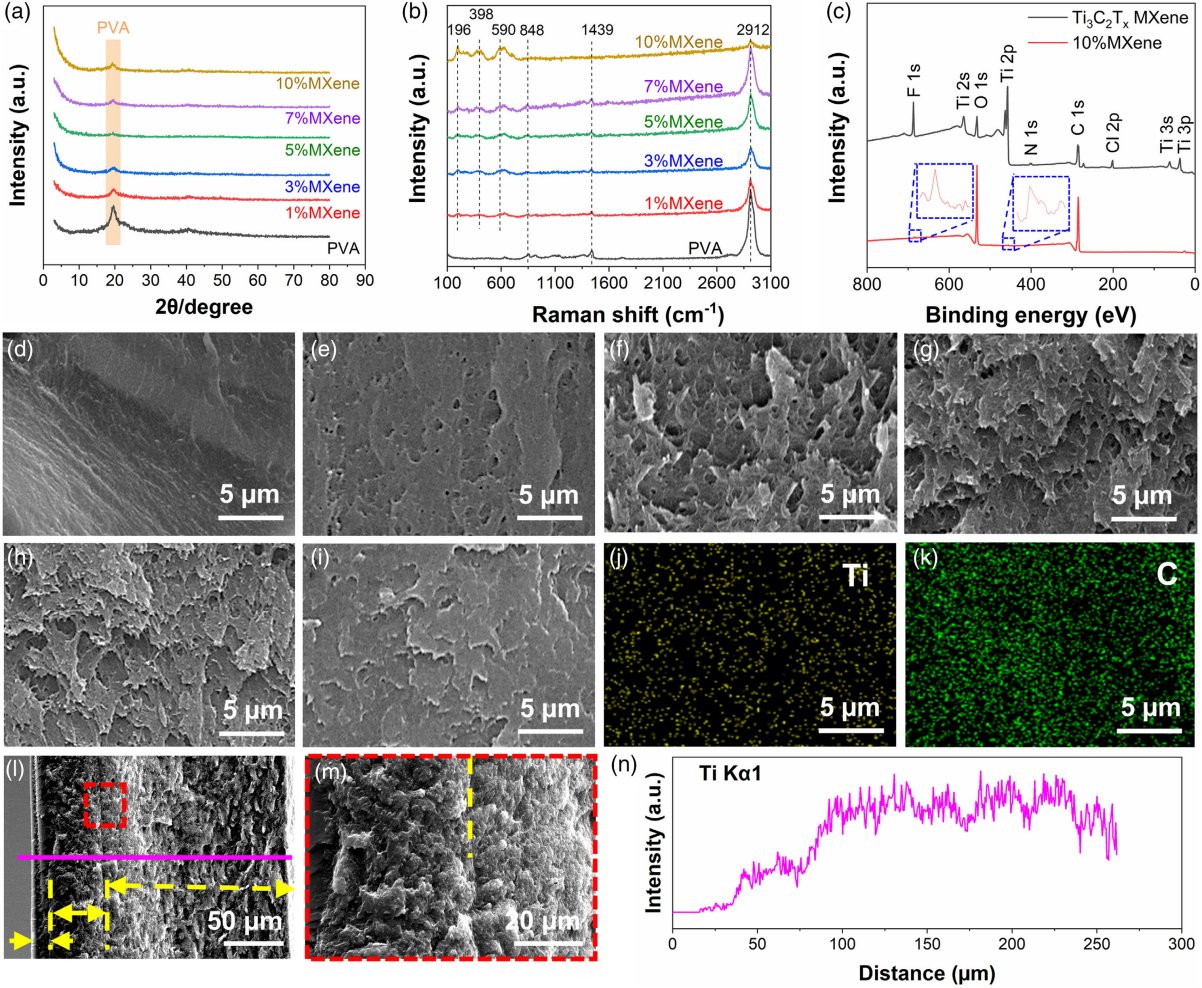
Characterization of single‐layer and three‐layer MXene/PVA films. a) X‐Ray diffraction (XRD) patterns and b) Raman spectra of PVA and composite films. c) X‐Ray photoelectron spectroscopy (XPS) survey spectra of pure MXene and 10% MXene. Cross‐sectional scanning electron microscope (SEM) images of d) PVA, e) 1% MXene, f) 3% MXene, g) 5% MXene, h) 7% MXene, and i) 10% MXene. j,k) Elemental mapping of 10% MXene. l,m) Cross‐sectional SEM images and n) energy‐dispersive X‐Ray spectroscopy (EDS) line scanning of M10‐5‐0 three‐layer gradient structure. Yellow dotted lines indicate the interface between two layers.

The microstructure and morphology of MXene/PVA films are confirmed by X‐Ray diffraction (XRD), X‐Ray photoelectron spectroscopy (XPS), and Raman spectrometers. **Figure** **2**a exhibits the XRD patterns of single‐layer Ti_3_C_2_T_*x*_/PVA composite films with different MXene contents. The diffraction peak at 2*θ* = 19.5° represents PVA phase which is consistent with reported work.^[^ ^23^, ^26^ ^]^ With higher MXene content, the intensity of PVA peak turns weaker. Due to the hydrogen bond between MXene and PVA, the crystallization of PVA during drying process may be disturbed leading to decreased crystallization degree. Specifically, the main diffraction peak of Ti_3_C_2_T_*x*_ MXene at 2*θ* = 6.2° disappears, which may be caused by the low filler content of MXene. Similarly, in a reported work, the MXene diffraction peak is almost unseen in MXene/PVA foam.^[^ ^23^ ^]^ Additionally, the intensity of the diffraction peak of MXene in MXene/PVA composite film becomes weaker with decreased MXene content. When MXene content reaches 40 wt%, the MXene peak can hardly be observed.^[^ ^26^ ^]^ Therefore, it is reasonable in this work that the MXene diffraction peak disappears considering that the maximum MXene content is only ≈10 wt%. Figure 2b reveals the Raman spectra of Ti_3_C_2_T_*x*_/PVA composite films. The peaks at Raman shift of 196, 398, and 590 cm^−1^ represent Ti_3_C_2_T_*x*_ MXene, which is in agreement with the reported article.^[^ ^9^ ^]^ Moreover, the peaks at Raman shift of 848, 1439, and 2912 cm^−1^ represent PVA, which is well matched with published research.^[^ ^27^ ^]^ Interestingly, the peak of TiO_2_ (located at Raman shift of ≈150 cm^−1^) is absent, indicating that MXene survived the drying process since TiO_2_ is the oxidation product of Ti_3_C_2_T_*x*_ MXene. Figure 2c shows the XPS survey spectra of pure Ti_3_C_2_T_*x*_ MXene and 10% MXene. Ti, C, O, and F elements are present in both pure MXene and 10% MXene, indicating the successful introduction of MXene into composite films. High‐resolution XPS spectra of Ti, C, O, and F elements are shown in Figure S3, Supporting Information, which further confirms the existence of these elements. In 10% MXene, the peak intensity of Ti and F is quite weak, which can be a support to the view that MXene is wrapped by PVA since XPS can only detect ≈10 nm in depth. The peaks of C and O become stronger due to the large amount of C and O in PVA.

Figure 2d–i is the cross sections of pure PVA and the composite films with different MXene contents. The cross section of pure PVA film is smooth, while the cross sections of composite films show zigzag pullout. It is known that MXene has good mechanical strength, which can act as strengthen phase in polymer matrix.^[^ ^28^ ^]^ In other words, MXene will bear most of the applied tensile force before cracking. The zigzag pullout is a proof of crack propagation. Afterward, cracked MXene can be pulled out from the matrix together with the surrounding PVA due to the strong hydrogen bond between them.^[^ ^29^, ^30^ ^]^ Thus, the as‐observed zigzag pullout is formed. Figure 2g,k is the elemental mapping of cross section of 10% MXene. Ti and C are uniformly distributed, indicating the uniform distribution of MXene in PVA. The hydrogen bond between the functional groups of MXene and hydroxide radical of PVA is the main reason for the homogeneous distribution. In addition, the cross section of M10‐5‐0 is also observed as exhibited in Figure 2l,m. The interface between layers can be clearly seen, which indicates a close contact between layers. In addition, the different thicknesses of each layer marked by yellow arrows in Figure 2l are due to the different amounts of cast mixtures. Figure 2n is the energy‐dispersive X‐Ray spectroscopy (EDS) line‐scanning result of Ti element. Three steps can be observed from the curve corresponding to the three layers with different contents of MXene. Therefore, the facile process of repeating casting–drying is an effective method to fabricate multilayer films.

### 2.2 EM Wave Absorption Performance of Single‐Layer Ti_3_C_2_T_*x*_/PVA Composite Films

**Figure 3:**
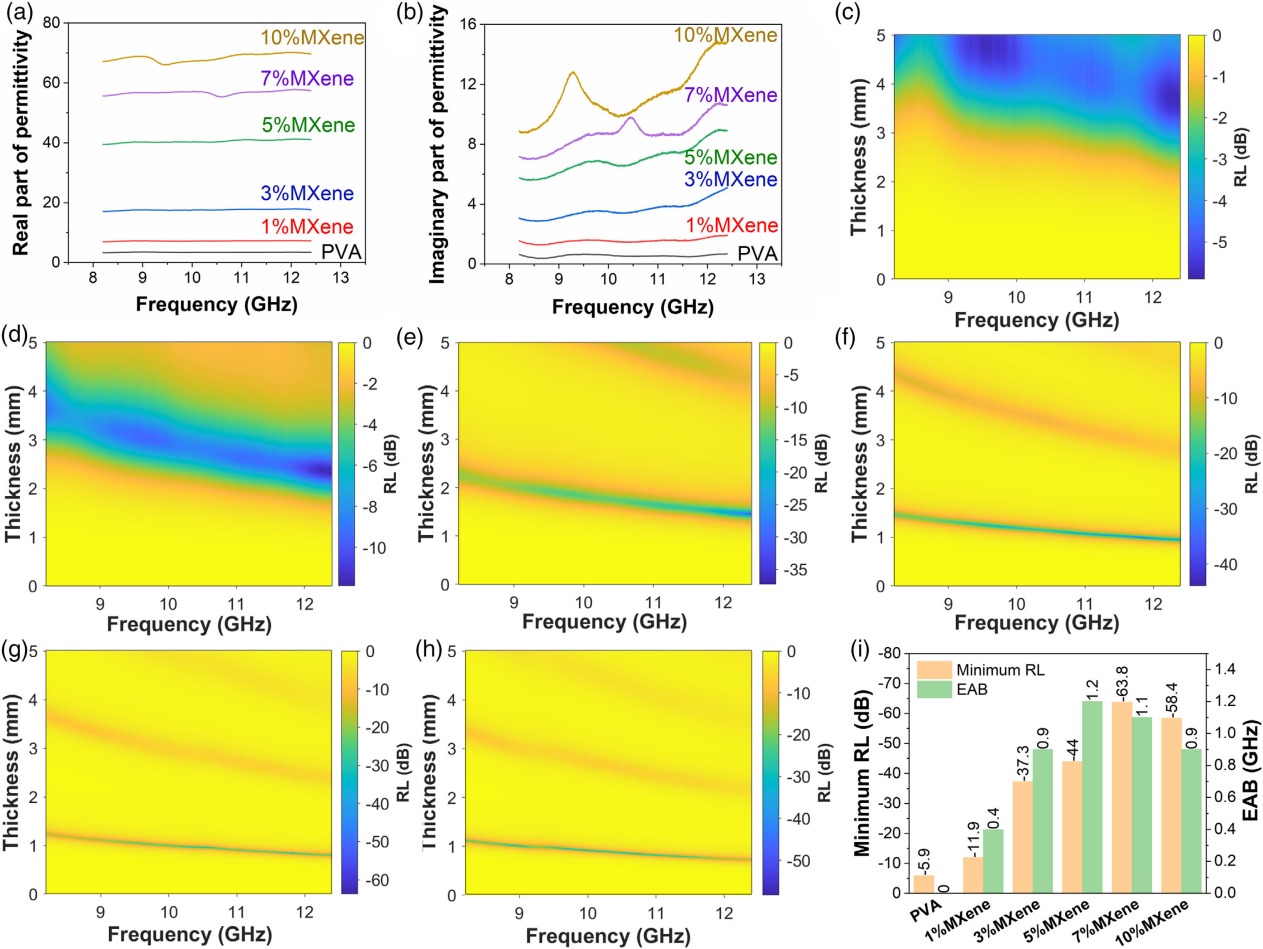
Permittivity and reflection loss (RL) of single‐layer films. a) Real and b) imaginary part of permittivity of single‐layer films as function of frequency. RL of c) pure PVA, d) 1% MXene, e) 3% MXene, f) 5% MXene, g) 7% MXene, and h) 10% MXene as function of thickness and frequency. i) Summary of minimum RL and corresponding effective absorbing bandwidth (EAB) for single‐layer films.

EM wave absorption performance is indicated by RL, which is calculated based on metal back‐panel model as described in the following equations.^[^ ^23^ ^]^

(1) $$Z_{\text{in}}=\sqrt{\frac{\mu_{r}}{\varepsilon_{r}}}\tanh\left(j\frac{2\pi fd}{c}\sqrt{\mu_{r}\varepsilon_{r}}\right)$$

(2) $$\text{RL}=20\;\log_{10}\left|\frac{Z_{\text{in}}-1}{Z_{\text{in}}+1}\right|$$

where $Z_{\text{in}}$ is the incident impedance, $\mu_{r}$ is the complex permeability, $\varepsilon_{r}$ is the complex permittivity, *f* is frequency of incident wave, *d* is the sample thickness, and *c* is the velocity of light. Since there is no magnetic phase in this work, $\mu_{r}$ is always equal to 1. **Figure** **3**a,b shows the real and imaginary part of the complex permittivity of Ti_3_C_2_T_*x*_/PVA composite films as a function of frequency. It can be observed that both real and imaginary part of permittivity increase with increasing MXene content. PVA as an EM wave transparent matrix has low permittivity, which is ≈3.4 for real part and ≈0.5 for imaginary part. MXene, as is known, has metallic conductivity, so the permittivity should be similar with metals, which are very high. Therefore, the permittivity of the composite increases with increasing MXene content. Figure 3c–h shows the RL of each Ti_3_C_2_T_*x*_/PVA composite film as a function of frequency and thickness, and Figure 3i shows the minimum RL of each composite film as well as the corresponding EAB. With elevated MXene content, the minimum RL decreases. Basically, the lower the RL, the better the EM absorption performance. For example, RL = −10 dB means 90% of the incident wave is absorbed, while RL = −20 dB means 99% of the incident wave is absorbed. For pure PVA, the minimum RL is −5.9 dB at 12.4 GHz and 3.69 mm sample thickness. For 7% MXene, the minimum RL reaches −63.8 dB at 11.8 GHz and 0.84 mm sample thickness. This vast improvement of EM absorption performance is mainly ascribed to strong cancellation between the first and second reflected wave, which will be discussed in the mechanism section. However, for 10% MXene, the minimum RL rises to −58.4 dB at 9.87 GHz and 0.93 mm sample thickness. The slight degradation of EM performance should be caused by the too large real part of permittivity, which will lead to over strong reflection of incident wave. For 1% MXene, 3% MXene, and 5% MXene, the minimum RL is −11.9 dB at 12.4 GHz and 2.35 mm, −37.3 dB at 12.4 GHz and 1.46 mm, −44 dB at 11.9 GHz and 0.99 mm, respectively.

Even though the minimum RL could reach −63.8 dB which means a rather strong absorption ability, the EAB of these single‐layer films is quite narrow due to that the main absorbing mechanism is cancellation. This will also be discussed in mechanism section. As can be seen from Figure 3i, the largest EAB is only 1.2 GHz for 5% MXene, which is far away from covering the whole X‐band (4.2 GHz width). Therefore, further structure design is necessary.

### 2.3 EM Wave Absorption Performance of Multilayer Ti_3_C_2_T_*x*_/PVA Composite Films

RL of multilayer material can be calculated based on transition line theory and metal back‐panel model. Suppose there are n layers in multilayer structure. The outermost layer which is close to air is numbered as 1st layer, while the innermost layer which is close to the metal back panel is numbered as *n*th layer. For the *i*th layer (*i* = 1, 2, …, n), the incident impedance $Z_{i}$ can be calculated by following equation.^[^ ^10^ ^]^

(3) $$Z_{i}=\eta_{i}\frac{Z_{i+1}+\eta_{i}\tanh\left(\gamma_{i}d_{i}\right)}{\eta_{i}+Z_{i+1}\tanh\left(\gamma_{i}d_{i}\right)}$$

where $Z_{i+1}$ is the incident impedance of the (*i* + 1)th layer, $\eta_{i}$ is the intrinsic impedance of the *i*th layer, $\gamma_{i}$ is the complex propagation constant of the *i*th layer, $d_{i}$ is the thickness of the *i*th layer. The intrinsic impedance and complex propagation constant can be calculated as the following formula.

(4) $$\eta_{i}=\eta_{0}\sqrt{\frac{\mu_{\text{ri}}}{\varepsilon_{\text{ri}}}}$$

(5) $$\gamma_{i}=\frac{j2\pi f}{c}\sqrt{\mu_{\text{ri}}\varepsilon_{\text{ri}}}$$

where $\eta_{0}$ is the intrinsic impedance of air ($\eta_{0}$ = 1), $\mu_{\text{ri}}$ is the relative complex permeability of the *i*th layer, $\varepsilon_{\text{ri}}$ is the relative complex permittivity of the *i*th layer, *f* is the frequency of incident wave, *c* is the velocity of light, and *j* is the imaginary unit. Since the incident impedance of metal back panel is 0 ($Z_{n+1}$ = 0), according to Equation (3), the incident impedance of the *n* th layer is

(6) $$Z_{n}=\eta_{n}\tanh\left(\gamma_{n}d_{n}\right)$$

By iteration, the incident impedance of the 1st layer can be obtained

(7) $$Z_{1}=\eta_{1}\frac{Z_{2}+\eta_{1}\tanh\left(\gamma_{1}d_{1}\right)}{\eta_{1}+Z_{2}\tanh\left(\gamma_{1}d_{1}\right)}$$

Finally, according to Equation (2), RL of the multilayer structure can be achieved

(8) $$\text{RL}=20\;\log_{10}\left|\frac{Z_{1}-1}{Z_{1}+1}\right|$$

In this work, to get the simplest gradient structure, three‐layer model is selected. Pure PVA layer is selected as the impedance matching layer due to that it has the closest permittivity with air. The 10% MXene is chosen as the reflective layer since it has the strongest reflective and lossy ability among the fabricated single composite films. Regarding the lossy layer, it is known that the permittivity should sit between impedance matching layer and reflective layer. However, what is the best permittivity of lossy layer and how it affects the EM absorbing performance of the gradient structure are still unclear and rarely studied in published articles. Therefore, the RL of a series of three‐layer gradient structures with different lossy layers including 1% MXene, 3% MXene, 5% MXene, and 7% MXene are calculated and compared. It is found that M10‐5‐0 has the lowest RL (−74.8 dB) and EAB covers the whole X‐band. EAB of M10‐3‐0 and M10‐7‐0 can also covers the entire X‐band, but minimum RL (−15–−25 dB) is significantly elevated. EAB of M10‐1‐0, however, cannot cover the whole X‐band.

**Figure 4:**
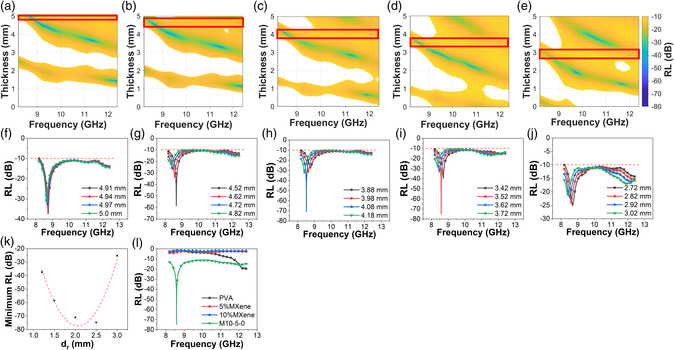
The 3D charts of RL of M10‐5‐0 as function of frequency and lossy layer thickness with reflective layer thickness of a) 1.2 mm, b) 1.5 mm, c) 2 mm, d) 2.5 mm, and e) 3 mm. The red boxes indicate the area where EAB covers the whole X‐band. f–j) Corresponding 2D charts of (a–e) in the range of the red boxes. k) Summary of relationship between minimum RL of (f–j**)** and reflective layer thickness *d*_r_. l) RL of M10‐5‐0 and component single‐layer films as function of frequency with the same thickness of 10.02 mm. Impedance matching layer thickness is 4 mm for all charts here.

**Figure** **4**a–e represents the RL of M10‐5‐0 with different reflective layer thickness (*d*_r_) as a function of frequency and lossy layer thickness (*d*_l_), which are calculated from permittivity of the single layers shown in Figure 3a,b through the transition line theory described previously. Here, impedance matching layer thickness (*d*_*i*_) is set as 4 mm for all cases according to Figure 3c, which shows the best RL around 4 mm. Only the area where RL < −10 dB is visible in these drawings. The red boxes suggest the optimal *d*_l_ range where EAB covers the whole X‐band. It can be observed that the location of the red boxes shifts down with increased reflective layer thickness. That means with increasing *d*_r_, the optimal range of *d*_l_ is decreased. To show the minimum RL in these red boxes, line charts are drawn as Figure 4f–j corresponding to Figure 4a–e. Specifically, in Figure 4i where *d*_r_ = 2.5 mm, the minimum RL could reach −74.8 dB and at the same time the EAB could cover the whole X‐band. Figure 4k summarizes the relationship between minimum RL in Figure 4f–j and *d*_r_. It can be seen that the minimum RL first decreases and then increases with elevated *d*_r_, which means *d*_r_ has an optimal point that can minimize the minimum RL. Figure S4, Supporting Information, shows that *d*_*i*_ also has an optimal value that can minimize the minimum RL, but the range of *d*_*i*_ (3.9–4.4 mm) that could make sure EAB cover the whole X‐band is much narrower than that of *d*_r_ (1.2–3 mm). In addition, the difference of minimum RL caused by *d*_*i*_ is much smaller than that caused by *d*_r_. To demonstrate that the outstanding EM absorbing performance of M10‐5‐0 benefits from the gradient structure rather than the accumulation of thickness, RL of the componential single layers with the same thickness as the total thickness of M10‐5‐0 is calculated. As presented in Figure 4l, pure PVA, 5% MXene, and 10% MXene, corresponding to the impedance layer, lossy layer, and reflective layer of M10‐5‐0, all have very poor absorbing performance even with the thickness of 10.02 mm, which is the total thickness of M10‐5‐0. In general, M10‐5‐0 possesses excellent absorbing ability with minimum RL of −74.8 dB and a broad EAB covering the whole X‐band owing to the good impedance matching caused by designed gradient multilayer structure.

The performance of M10‐5‐0 is compared to reported microwave absorbing materials. There are many carbon‐based and magnetic absorbers beyond MXene.^[^ ^31^, ^32^, ^33^, ^34^, ^35^ ^]^ As an advanced carbon‐based absorbing agent, carbon nanotubes @ carbon fiber was fabricated via in situ catalytic grown. The minimum RL is −53 dB and corresponding EAB is 1.2 GHz.^[^ ^31^ ^]^ A typical magnetic absorber, Co/C nanocomposite, was derived from pyrolysis of metal organic framework. The minimum RL is −30.31 dB with an EAB of 4.93 dB.^[^ ^32^ ^]^ The performance of some other MXene‐based absorbers is shown in Table S1, Supporting Information. Through comparison with these data, the performance of the gradient structure in this work is competitive. A shortage of this three‐layer structure is the relatively larger thickness, which needs further improvement in our future work such as involving heterogeneous interface to increase polarization loss and magnetic phase to induce magnetic loss and so on. The importance of this work is to demonstrate the effectiveness of the three‐layer gradient structure and show some rules between the performance of the gradient structure and componential layers as the first step.

**Figure 5:**
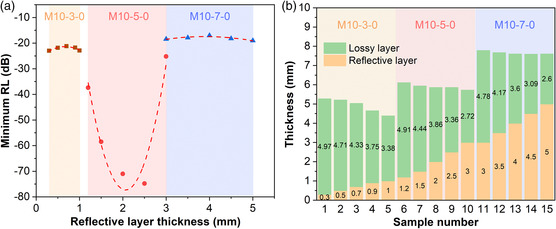
a) Relationship between minimum RL and reflective layer thickness for different three‐layer structures. b) Reflective layer thickness and corresponding minimum lossy layer thickness for each sample.

**Figure** **5**a exhibits the relationship between minimum RL and *d*_r_ for M10‐3‐0 and M10‐7‐0, and M10‐5‐0 is also presented to detect the impact of lossy layer. Detailed RL of M10‐3‐0 and M10‐7‐0 can be found in Figure S5 and S6, Supporting Information. For M10‐3‐0, the range of *d*_r_ that could promise the EAB covering the whole X‐band is 0.3–1 mm, while that for M10‐5‐0 is 1.2–3 mm and for M10‐7‐0 is 3–5 mm (thickness larger than 5 mm is out of scope in this work). Therefore, the optimal thickness range of reflective layer is increasing with increasing permittivity of lossy layer. A reasonable explanation is larger permittivity of lossy layer means better impedance matching between lossy layer and reflective layer, so less reflected wave on the interface between lossy layer and reflective layer. To achieve cancelation on the interface, second reflected wave (reflected by the interface between reflective layer and metal back panel) should also be weakened. Therefore, a thicker reflective layer is needed to prolong the wave path and weaken the second reflected wave. Additionally, both M10‐3‐0 and M10‐7‐0 have higher minimum RL than M10‐5‐0. This should be due to the relatively larger impedance mismatching between impedance matching layer and lossy layer for M10‐7‐0 and between lossy layer and reflective layer for M10‐3‐0. In other words, the overall impedance matching is the best when permittivity of lossy layer is close to the average permittivity of impedance matching layer and reflective layer.

Figure 5b reveals the thickness of reflective layer and the corresponding minimum thickness of lossy layer that could make sure EAB covers the whole X‐band. In each three‐layer model, the minimum d_l_ decreases with increasing *d*_r_, while the sum of *d*_l_ and *d*_r_ almost keeps stable. Moreover, the sum of *d*_l_ and *d*_r_ increases stepwise from M10‐3‐0 to M10‐5‐0 further to M10‐7‐0. That means total thickness of three‐layer structure increases with increasing permittivity of lossy layer since the thickness of impedance matching layer is kept unchanged as 4 mm.

Figure S7, Supporting Information, shows RL of M10‐1‐0 with different di and dr. Only the area where RL < −10 dB is visible in the drawings. It can be seen that no di and dr could make the EAB cover the whole X‐band. A possible explanation for this is that the permittivity of lossy layer is much too close to the permittivity of impedance matching layer and the lossy layer actually acts as an impedance matching layer to some extent. As a consequence, the gradient structure cannot fully work, and EAB is not fully broadened.

To sum up for this section, for a three‐layer gradient absorbing structure, the lossy layer should possess a permittivity close to the average permittivity of impedance matching layer and reflective layer so that the structure could achieve the strongest absorbing ability. If a thinner total thickness is desired, the permittivity of lossy layer should be decreased but not too close to the permittivity of impedance matching layer, and the minimum RL will increase as a sacrifice.

### 2.4 EM Wave Absorption Mechanism of Gradient Multilayer Structure

As can be observed in Figure 3, the minimum RL of single‐layer films with relatively higher MXene content can also reach a very low level even though the impedance matching between air and these films is rather poor. The strong absorption ability of these single films is derived from cancellation of first reflected wave, which is reflected by the upper surface of absorber and second reflected wave, which is reflected by the lower surface of absorber. Therefore, very proper thickness, frequency, and permittivity are required to achieve intense cancellation. This is the reason why EAB of these single films is very narrow.

**Figure 6:**
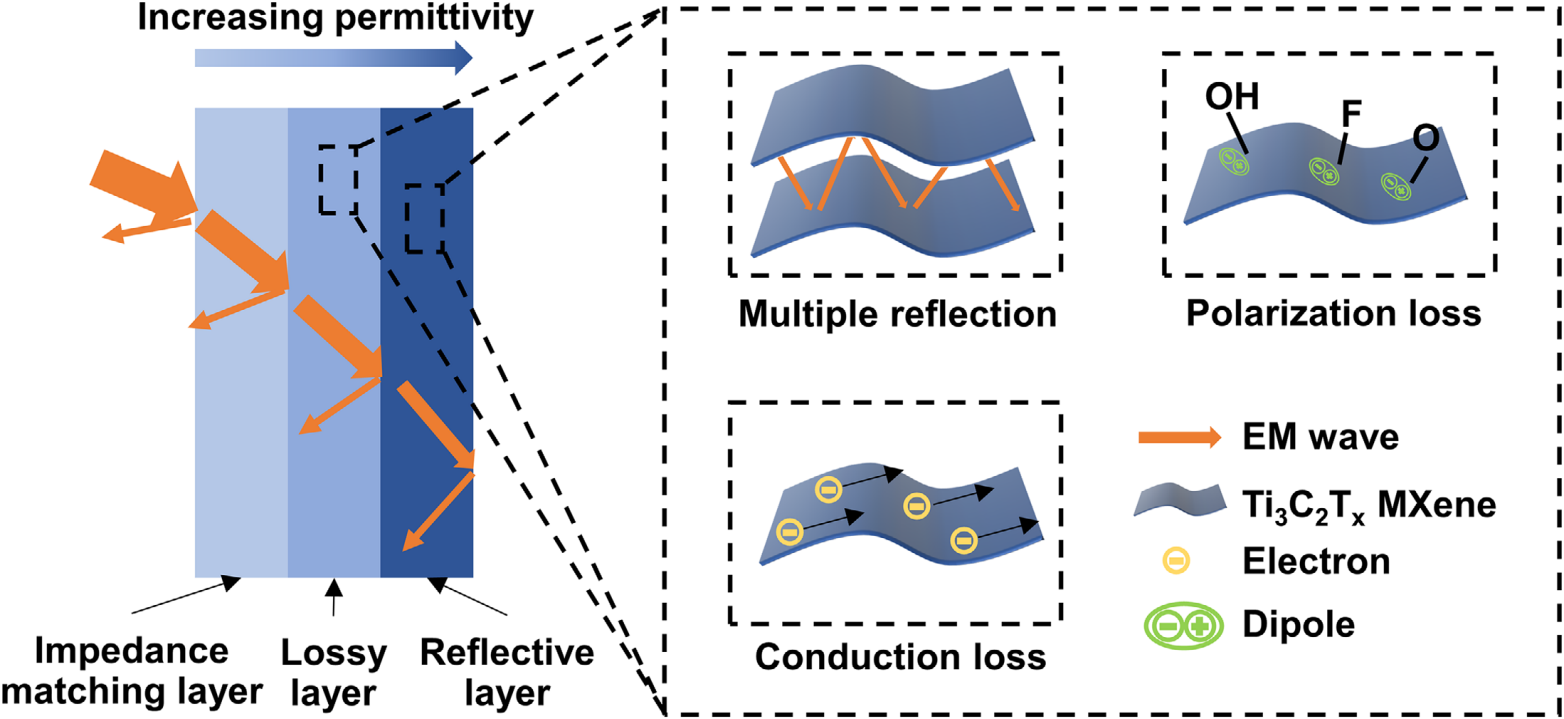
Electromagnetic (EM) absorbing mechanism of three‐layer gradient structure.

Distinguished from single‐layer absorber, the outstanding performance of gradient multilayer absorber benefits from improved impedance matching, which is indicated in **Figure** **6**. Pure PVA has good impedance matching with air due to their closed permittivity. When EM wave incidents into multilayer absorber, just a small portion of the incident wave will be reflected since the first layer is pure PVA. Then, most of the incident wave could reach lossy layer. If the wave comes from air, the impedance matching will be poor. However, in this case, the incident wave to lossy layer is from PVA layer, and the permittivity difference between PVA layer and lossy layer is smaller than the difference between air and lossy layer. As a result, the impedance matching is improved and most of the incident wave from PVA layer could penetrate into lossy layer and be consumed by Ti_3_C_2_T_*x*_ MXene via multiple reflection, conduction loss, and polarization loss.^[^ ^19^, ^23^, ^36^ ^]^ After that, the residual EM wave could reach reflective layer. Similarly, since the incident wave to reflective layer comes from lossy layer, the impedance matching is significantly improved because of the smaller permittivity difference between lossy layer and reflective layer compared with the difference between air and reflective layer. As a consequence, most of the incident wave to reflective layer could go into it. Due to the amount of Ti_3_C_2_T_*x*_ MXene in reflective layer is greater than in lossy layer, the consumption of EM wave is even more intense. Therefore, just very limited EM wave could pass through the gradient structure. Inevitably, there are weak reflected waves on surface of each layer, so thickness optimization is still needed to obtain cancellation of these reflected waves. However, the cancellation is no longer the predominant role. For situations where cancellation is weak or absent, the EM absorption ability is still considerable. Therefore, the EAB is broadened.

## 3 Conclusion

A three‐layer gradient structure is established for strong and broadband microwave absorption. The gradient structure is built via repeating casting–drying method using different amount of Ti_3_C_2_T_*x*_ MXene and PVA for each layer. The minimum RL could reach −74.8 dB with EAB covering the entire X‐band. Unlike the single‐layer structure which mainly relies on cancellation, the gradient structure primarily depends on optimized impedance matching to realize remarkable microwave absorbing performance. Moreover, RL of the gradient structure could achieve the lowest value when the permittivity of lossy layer is close to the average permittivity of impedance matching layer and reflective layer. In addition, the lower the permittivity of lossy layer, the thinner the total thickness of the gradient structure. These conclusions may be promoted beyond MXene and PVA to any other absorbing fillers and microwave‐transparent matrix. This work demonstrates the effectiveness of a simple three‐layer gradient structure as a strong and broadband microwave absorber and provides rules to design suitable gradient structures to satisfy different applications.

## 4 Experimental Section

4.1

4.1.1

##### Materials

TiC (2–4 μm, 99%), Ti (300 mesh, 99.99%), and LiF (analytical reagent (AR), 99%) powders were purchased from Aladdin Reagent Co., Ltd (China). Al (300 mesh, 99.7%) powder was purchased from Zhongnuo Advanced Material (Beijing) Technology Co., Ltd. HCl (AR, 38%) was purchased from Sinopharm Chemical Reagent Co., Ltd (China). PVA (AR, Mw. 89 000–98 000) was purchased from Sigma‐Aldrich. All chemical reagents were used without further treatment.

##### Synthesis of Ti_3_AlC_2_

TiC, Ti, and Al with a mole ratio of 1.8:1:1 were mixed by a 3D powder mixer for 24 h. Then, the mixture was put in an alumina crucible and heated to 1450 °C for 2 h in an argon atmosphere to sinter Ti_3_AlC_2_. The derived bulk ceramic was crushed and sieved through a 300 mesh screen.

##### Synthesis of Few‐Layer Ti_3_C_2_T_x_ Colloid

First, 5 g of LiF was added into 100 mL HCl and magnetic stirred at 500 rpm for 10 min to fully dissolve LiF. Then, 5 g of Ti_3_AlC_2_ was slowly added to the solution. Afterward, the mixture was kept at 45 °C for 24 h with continuous stirring to selectively etch the Al layer of Ti_3_AlC_2_. Then, the reaction product was repeatedly washed with deionized water by centrifugation at 4500 rpm for 5 min until pH of the supernatant reached 6–7 and the sediment was collected. The 100 mL deionized water was added into the sediment, followed by sonication in ice water bath for 60 min with argon as the protective gas. The derived product was centrifuged at 3500 rpm for 60 min and the black supernatant was collected, which is the few‐layer Ti_3_C_2_T_*x*_ colloid. The concentration of the colloid was measured by freeze‐drying a certain volume of the colloid, and the measured concentration was about 15 mg mL^−1^.

##### Synthesis of Ti_3_C_2_T_x_/PVA Composite Films

First, 10 g of PVA powder was slowly added to 90 g deionized water and magnetically stirred for 3 h at 95 °C to prepare 10 wt% PVA solution. Afterward, different amount of Ti_3_C_2_T_*x*_ colloid was added into 10 g of the PVA solution. The mass ratio of Ti_3_C_2_T_*x*_ to PVA is 1:100, 3:100, 5:100, 7:100, and 10:100, respectively. Then, the mixtures were magnetically stirred at 500 rpm for 4 h. Subsequently, the mixtures were cast into plastic Petri dishes and dried at 60 °C for 48 h to get the single‐layer Ti_3_C_2_T_*x*_/PVA composite films. PVA film was made by the same process without adding Ti_3_C_2_T_*x*_ colloid. Regarding the synthesis of multilayer composite film, taking M10‐5‐0 as an example, Ti_3_C_2_T_*x*_ and PVA mixture with the mass ratio of 10:100 was first cast into a Petri dish and dried at 60 °C for 48 h. Then, Ti_3_C_2_T_*x*_ and PVA mixture with mass ratio of 5:100 was cast into the same Petri dish and dried at 60 °C for 48 h. Finally, pure PVA was cast into that Petri dish and dried at 60 °C for 48 h.

##### Characterization

The microstructure of the films was confirmed by XRD (Haoyuan, DX‐2700BH), laser Raman spectrometer (Witec Alpha300, *λ* = 532 nm), and XPS (Thermo Scientific K‐Alpha, Al Kα radiation). Morphology was observed by a Sirion field‐emission SEM equipped with an EDS apparatus (Zates, X‐MAX 80) at an accelerating voltage of 15 kV. Scattering parameters of the films were measured using Agilent E5071C vector network analyzer via waveguide method in X‐band. The films were tailored into 10.16 × 22.86 mm and put in the rectangular waveguide. EM parameters were calculated using scattering parameters through Nicholson–Ross–Weir (NRW) theory.^[^ ^37^ ^]^

## Conflict of Interest

The authors declare no conflict of interest.

## Supporting information

Supplementary Material

## Data Availability

The data that support the findings of this study are available from the corresponding author upon reasonable request.
