# Supplementary material for: Gradient Multilayer Design of Ti3C2T x MXene Nanocomposite for Strong and Broadband Microwave Absorption
Source: Small Sci. 2022 May 21;2(7):2200018. doi: 10.1002/smsc.202200018 (PMC11935905; doi:10.1002/smsc.202200018)
Supplement: Supplementary file 1 — Supplementary Material [file SMSC-2-2200018-s001.pdf]

Supporting Information

Gradient multilayer design of $Ti_3C_2T_x$ MXene nanocomposite for strong and broadband microwave absorption

Yajun Zhang, Long Pan, Peigen Zhang, ZhengMing Sun**

Y. Zhang, Prof. L. Pan, Prof. P. Zhang, Prof. Z.M. Sun
Key Laboratory of Advanced Metallic Materials of Jiangsu Province
School of Materials Science and Engineering
Southeast University
Nanjing 211189 PR China
E-mail: panlong@seu.edu.cn (L. Pan), zmsun@seu.edu.cn (Z.M. Sun)

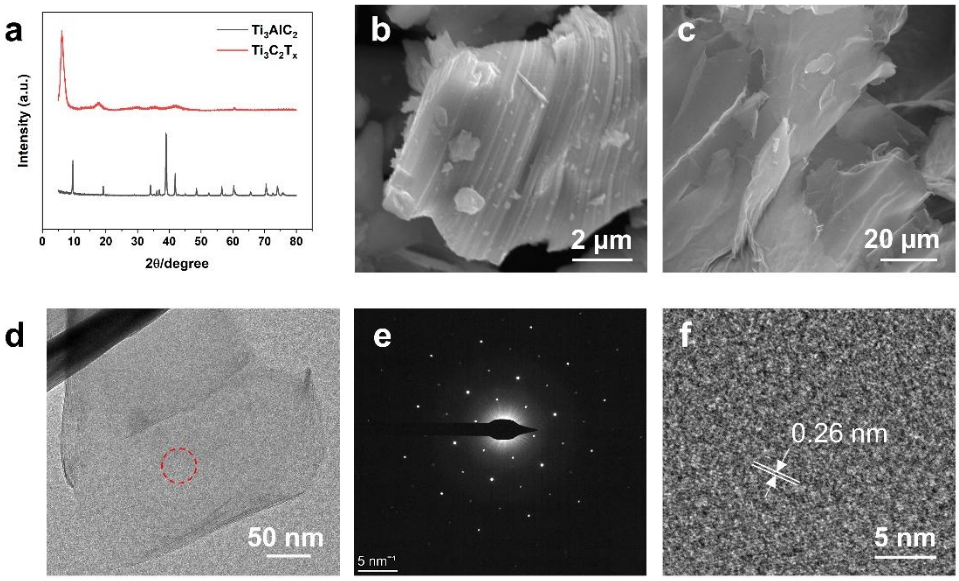

Figure S1. Characterization of MAX and MXene. **a** XRD patterns of Ti_3AlC_2 MAX and $Ti_3C_2T_x$ MXene. SEM images of **b** Ti_3AlC_2 MAX and **c** $Ti_3C_2T_x$ MXene. **d** TEM, **e** SAED and **f** HRTEM images of $Ti_3C_2T_x$ MXene.

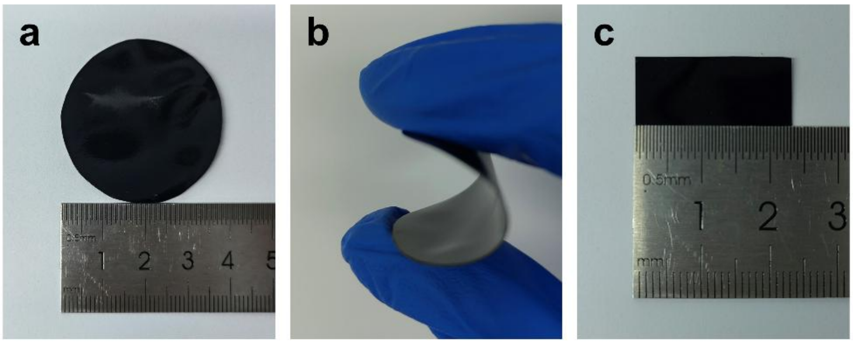

Figure S2. Photographs of the obtained 10%MXene composite film. **a** Original film peeled off from Petri dish with cut edge. **b** Bending film showing the flexibility. **c** Testing sample for scattering parameter measurement.

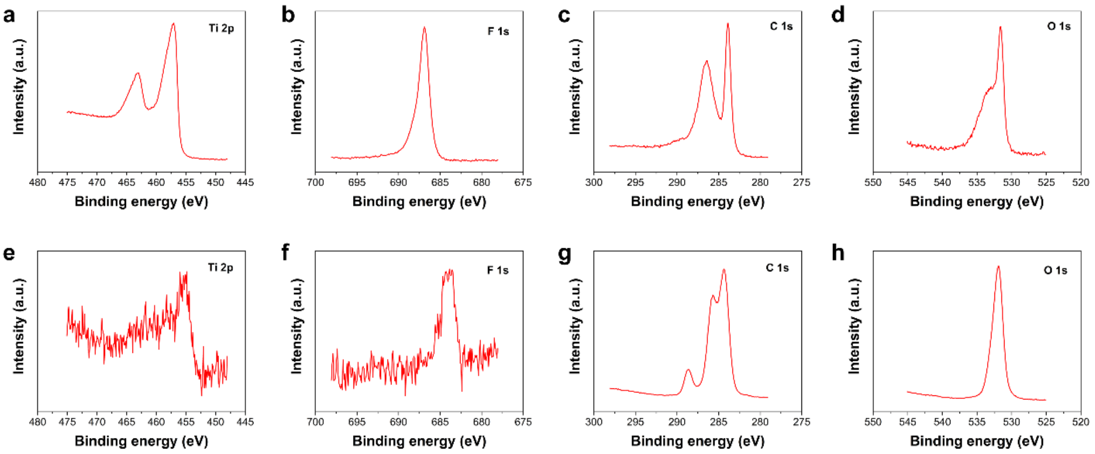

Figure S3. High-resolution XPS spectra of **a-d** pure $Ti_3C_2T_x$ MXene and **e-h** 10%MXene.

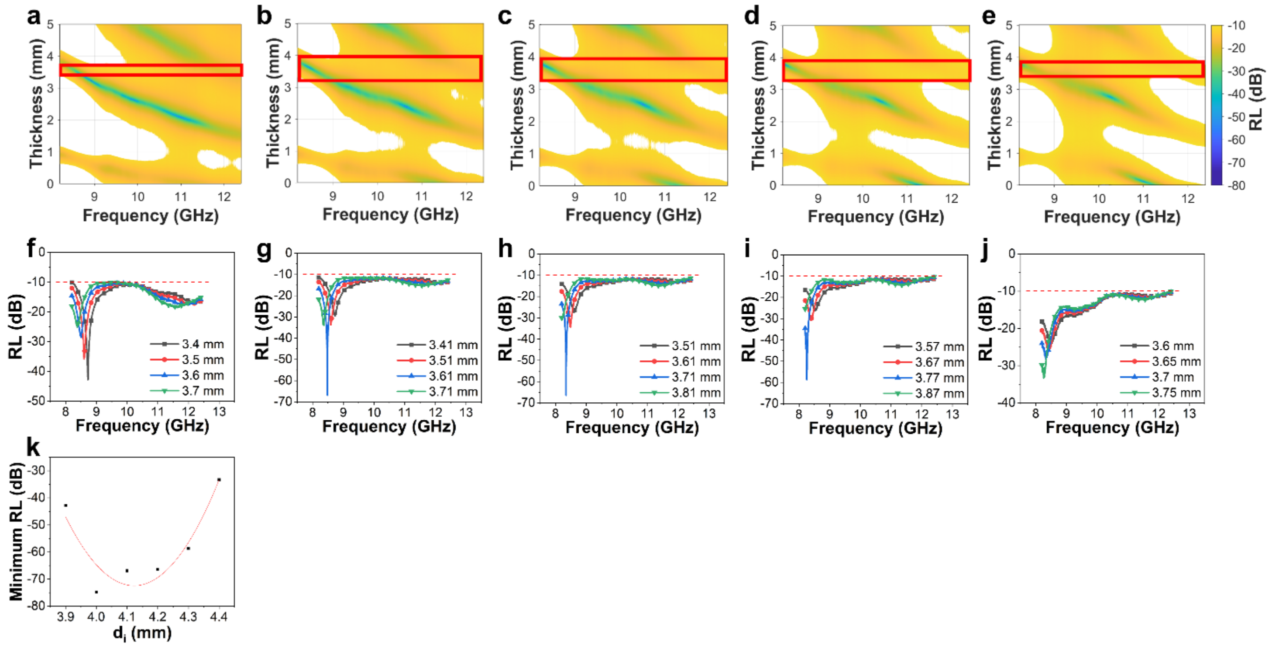

Figure S4. 3D charts of RL of M10-5-0 as function of frequency and lossy layer thickness with impedance matching layer thickness of **a** 3.9 mm, **b** 4.1 mm, **c** 4.2 mm, **d** 4.3 mm, **e** 4.4 mm. The red boxes indicate the area where EAB covers the whole X-band. **f-j** Corresponding 2D charts of **a-e** in the range of the red boxes. **k** Summary of relationship between minimum RL of **f-j** and impedance matching layer thickness d_i. Reflective layer thickness is 2.5 mm for all charts here.

Table S1. EM absorbing performance of reported $Ti_3C_2T_x$-based absorbing materials and this work.

**Absorber**	**Matrix**	**RL_{min} (dB)**	**EAB (GHz)**	**Tested frequency range (GHz)**	**Thickness (mm)**	**Ref.**
$Ti_3C_2T_x$/PANI	paraffin	-56.3	4.28	2-18	1.8	[1]
$Ti_3C_2T_x$/graphene	-	-23	4.2	8.2-12.4	3.2	[2]
$Ti_3C_2T_x$/cellulose	-	-43.4	4.5	2-18	2.5	[3]
Annealed $Ti_3C_2T_x$	wax	-45	2.8	8.2-12.4	1.85	[4]
Graphite/TiC/$Ti_3C_2T_x$	wax	-63	3.5	8.2-12.4	2.1	[5]
$Ti_3C_2T_x$/carbon sphere	wax	-54.7	1.1	8.2-12.4	4.8	[6]
TiO_2/$Ti_3C_2T_x$/Fe_3O_4	Wax	-57.3	2	8.2-12.4	1.9	[7]
Ni/$Ti_3C_2T_x$	PVDF	-52.6	3.7	8.2-12.4	3.0	[8]
$Ti_3C_2T_x$	PVA	-18.7	4.2	8.2-12.4	3.9	[9]
$Ti_3C_2T_x$(7-layer)	polymer	-26.1	4.2	8.2-12.4	7.0-7.5	[10]
$Ti_3C_2T_x$(3-layer)	PVA	-74.8	4.2	8.2-12.4	10.02	This work

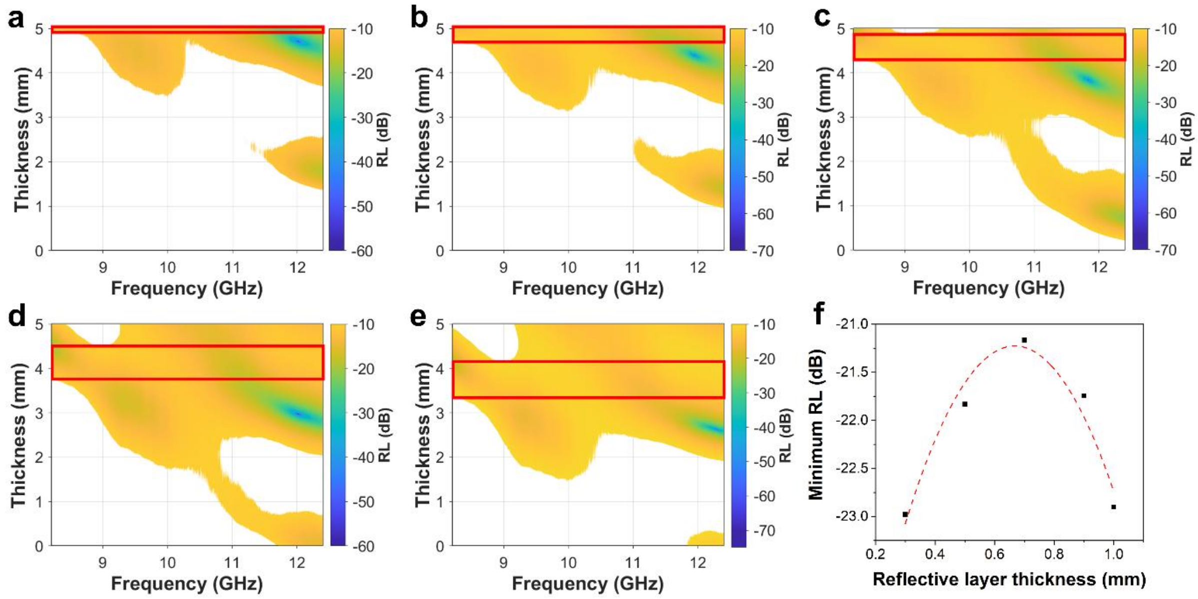

Figure S5. 3D charts of RL of M10-3-0 as function of frequency and lossy layer thickness with reflective layer thickness of **a** 0.3 mm, **b** 0.5 mm, **c** 0.7 mm, **d** 0.9 mm, **e** 1.0 mm. The red boxes indicate the area where EAB covers the whole X-band. **f** Summary of relationship between minimum RL in the red boxes and reflective layer thickness. Impedance matching layer thickness is 4 mm for all charts here.

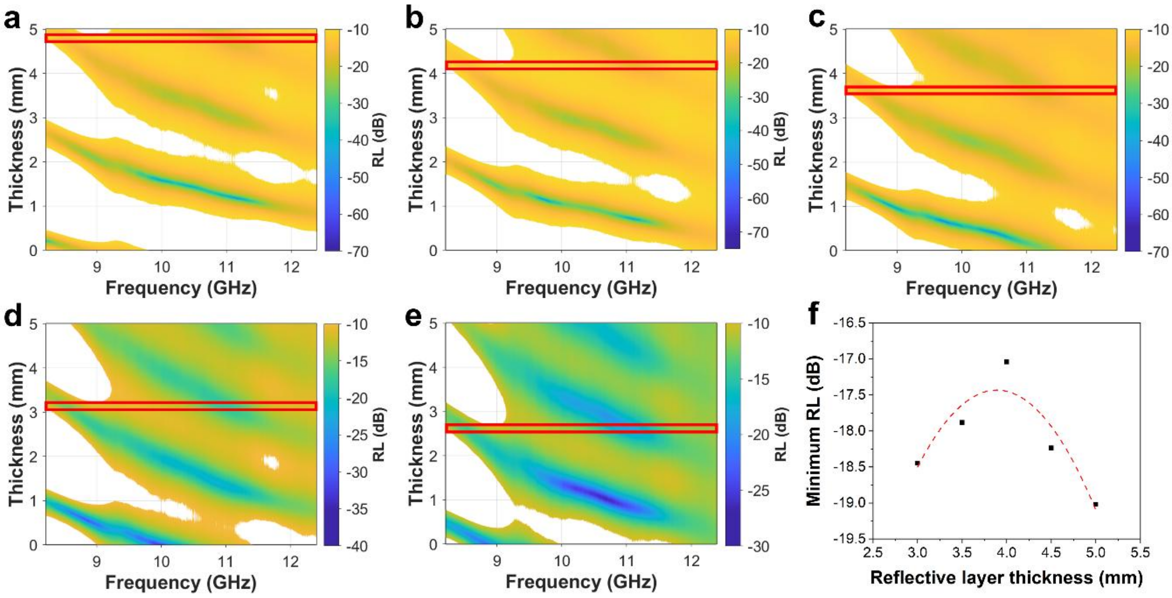

Figure S6. 3D charts of RL of M10-7-0 as function of frequency and lossy layer thickness with reflective layer thickness of **a** 3 mm, **b** 3.5 mm, **c** 4 mm, **d** 4.5 mm, **e** 5 mm. The red boxes indicate the area where EAB covers the whole X-band. **f** Summary of relationship between minimum RL in the red boxes and reflective layer thickness. Impedance matching layer thickness is 4 mm for all charts here.

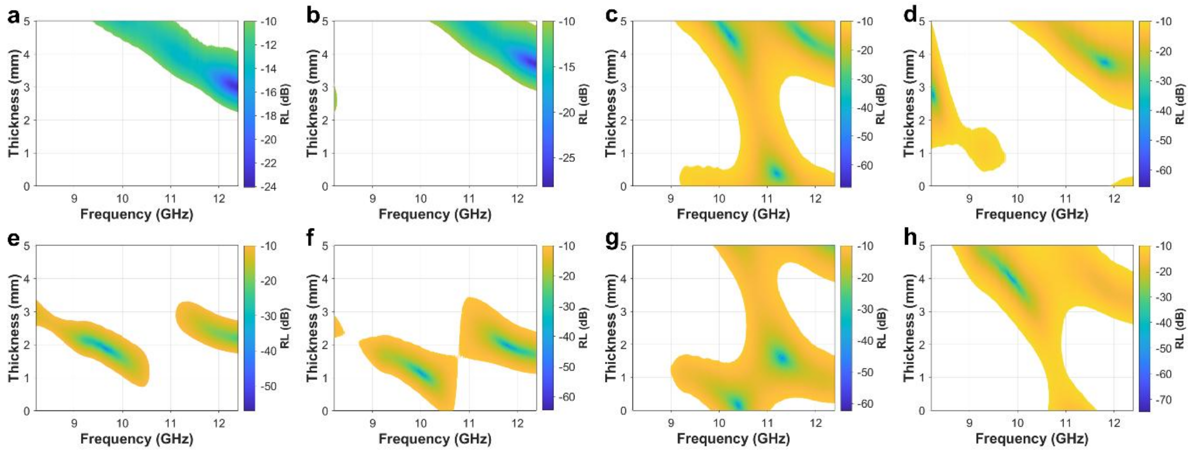

Figure S7. 3D charts of RL of M10-1-0 as function of frequency and lossy layer thickness with reflective layer thickness of **a** 0.5 mm, **b** 1.5 mm, **c** 2.5 mm, **d** 3.5 mm. Impedance matching layer thickness is 4 mm for **a-d**. 3D charts of RL of M10-1-0 as function of frequency and lossy layer thickness with impedance matching layer thickness of **e** 1 mm, **f** 2 mm, **g** 3 mm, **h** 5 mm. Reflective layer thickness is 2.5 mm for **e-h**. EAB of all charts here cannot cover the whole X-band.

References

[1] H. Wei, J. Dong, X. Fang, W. Zheng, Y. Sun, Y. Qian, Z. Jiang, Y. Huang, *Compos. Sci. Technol.* **2019,** *169*, 52-59.

[2] X. Li, X. Yin, C. Song, M. Han, H. Xu, W. Duan, L. Cheng, L. Zhang, *Adv. Funct. Mater.* **2018,** *28*.

[3] Y. Jiang, X. Xie, Y. Chen, Y. Liu, R. Yang, G. Sui, *Journal of Materials Chemistry C* **2018,** *6*, 8679-8687.

[4] M. Han, X. Yin, H. Wu, Z. Hou, C. Song, X. Li, L. Zhang, L. Cheng, *ACS Appl Mater Interfaces* **2016,** *8*, 21011-9.

[5] M. Li, M. Han, J. Zhou, Q. Deng, X. Zhou, J. Xue, S. Du, X. Yin, Q. Huang, *Advanced Electronic Materials* **2018,** *4*.

[6] B. Dai, B. Zhao, X. Xie, T. Su, B. Fan, R. Zhang, R. Yang, *Journal of Materials Chemistry C* **2018,** *6*, 5690-5697.

[7] P. Liu, Z. Yao, V. M. H. Ng, J. Zhou, L. B. Kong, K. Yue, *Composites Part A: Applied Science and Manufacturing* **2018,** *115*, 371-382.

[8] L. Liang, R. Yang, G. Han, Y. Feng, B. Zhao, R. Zhang, Y. Wang, C. Liu, *ACS Appl Mater Interfaces* **2020,** *12*, 2644-2654.

[9] H. Xu, X. Yin, X. Li, M. Li, S. Liang, L. Zhang, L. Cheng, *ACS Appl Mater Interfaces* **2019,** *11*, 10198-10207.

[10] B. Ji, S. Fan, S. Kou, X. Xia, J. Deng, L. Cheng, L. Zhang, *Carbon* **2021,** *181*, 130-142.
